# Subjective age and the association with intrinsic capacity, functional ability, and health among older adults in Norway

**DOI:** 10.1007/s10433-023-00753-2

**Published:** 2023-02-28

**Authors:** Ellen Melbye Langballe, Vegard Skirbekk, Bjørn Heine Strand

**Affiliations:** 1 Norwegian National Centre for Ageing and Health, Vestfold Hospital Trust, Tønsberg, Norway; 2 Department of Geriatric Medicine, Oslo University Hospital, Oslo, Norway; 3 Norwegian Institute of Public Health, Oslo, Norway

**Keywords:** Subjective age, Intrinsic capacity, Hearing, Vision, Incontinence

## Abstract

This study investigates the relationships between subjective age, intrinsic capacity, functional ability and health among Norwegians aged 60 years and older. The Norwegian Survey of Health and Ageing (NORSE) is a population-based, cross-sectional study of home-dwelling individuals aged 60–96 years in the former county of Oppland. Age- and sex-adjusted regression models were used to investigate the gap between subjective and chronological age and this gap’s association with self-reported and objectively measured intrinsic capacity (covering all six sub domains defined by WHO), health, and functional ability among 817 NORSE participants. The results show most participants felt younger than their chronological age (86.5%), while relatively few felt the same as their chronological age (8.3%) or older (5.2%). The mean subjective age was 13.8 years lower than mean chronological age. Participants with incontinence, poor vision, or poor hearing felt 3.1 [95% confidence interval (CI) (0.6, 5.5)], 2.9 [95% CI (0.2, 5.6)], and 2.9 [95% CI (0.3, 5.5)] years older, respectively, than participants without those conditions, whereas none of the following factors—anxiety, depression, chronic disease, Short Physical Performance Battery score, grip strength, cognition, or frailty—significantly had an impact on the gap. In line with prior research, this study finds that feeling considerably younger than one’s chronological age is common at older ages. However, those with poor hearing, poor vision, and incontinence felt less young compared to those not having these conditions. These relationships may exert undesirable effects on vitality and autonomy, which are considered key factors of intrinsic capacity and healthy ageing.

## Introduction

How old you feel defines your subjective age and has important relevance for social, health, and economic factors. One’s subjective age relates to social relationships and comparison groups influencing self-perceptions of age (Settersten and Hagestad 2015). It is contextual and can depend on which groups on compares oneself to (Sayag and Kavé 2022). A literature review concluded that the most frequent themes considered when assessing older adults’ self-perceived age were attitudes towards one’s own ageing, own well-being, stereotypes of ageing, ageing identities, the ageing body, and one’s future self-view (Hausknecht et al. 2020).

Chronologically older individuals tend to show a wider gap between their chronological and subjective ages, but that there is large individual variation (Shinan-Altman and Werner 2019). It is well-established that it is common for older adults to feel considerably younger than their chronological age (Pinquart and Wahl 2021) particularly if they have an active role in society (Rubin and Berntsen 2006; Gendron et al. 2018; Kwak et al. 2018; Stephan et al. 2018a, b). Being socially active, in good health and having more economic resources are associated with a younger subjective age relative to one’s chronological age (Skirbekk et al. 2019; Hajek and König 2020; Ye and Post 2020). A younger subjective age relates to a host of outcomes concerning activity and social participation, such as Internet use among people 65 years old or older (Seifert and Wahl 2018). This indicates that subjective age and feeling young can be important for healthy ageing.

### Subjective age

Health can have important implications for how old one feels and correlate with well-being (Kotter-Grühn et al. 2016). This important relationship can be reciprocal (Larkin 2013). Good health may promote a feeling of young subjective age, whereas the sense of feeling younger than one’s chronological age may induce a positive health behaviour (Aftab et al. 2022), and lead to increased work participation and more income and savings (Ye and Post 2020). A younger subjective age relative to one’s chronological age relates to better somatic and mental health, improved cognitive functioning, reduced hospitalization risks, and lower mortality (Stephan et al. 2018a, b; van Solinge and Henkens 2018; Hajek and König 2020; Schroyen et al. 2020; Ye and Post 2020). One study of 875 older women and men found that health as well as satisfaction with health accounted for one third of the variance in subjective age (Hubley and Russell 2009). Another study found that a younger subjective age was associated with a slower decline in functional health in a three-year follow-up study using the German Ageing Survey (Wettstein et al. 2021a, b). One Norwegian study found that wanting to be younger negatively related to life satisfaction and physical functioning over time, although variation in subjective age did not predict subsequent well-being or physical functioning (Veenstra et al. 2021). Moreover, depression is associated with both older subjective age and negative attitudes towards own ageing (Schönstein et al. 2021), whereas younger subjective age is associated with better subjective well-being and cognitive performance (Debreczeni and Bailey 2021).

Poor childhood health additionally relates to an older subjective age in adulthood and older age (Smith and Larkina 2021). Experiencing ageing-associated conditions such as grey hair, restricted mobility, and onset of chronic illness relatively early in life can lead to a feeling of being older, while conditions that are more temporary may not affect subjective age the same way (e.g. depression, viral infection, bodily aches, and exhaustion) (Leone and Hessel 2016; Agrigoroaei 2018; Stephan et al. 2018a, b; Sayag and Kavé 2022).

Particularly among older adults, a younger subjective age may help mitigate declining functional health (Wettstein et al. 2021a, b). Individuals who feel younger by a certain amount, but not more, have been found to have the highest levels of life satisfaction. This “optimal discrepancy” between subjective and chronological age widens across the adult age span—having an increasingly lower subjective age relative to chronological age relates to higher well-being as one grows older (Blöchl et al. 2021).

### Intrinsic capacity

The World Health Organization (WHO) has defined *healthy ageing* as an “ongoing process of developing and maintaining the functional ability that enables well-being in older age” (Beard et al. 2016). Functional ability is defined as determined by the interaction of a person’s intrinsic capacity (IC) and the environment. Whereas IC encompasses all physical and mental capacities, the environment in that context includes access to support that may facilitate functional ability and offer opportunities to exert control over that environment (Michel et al. 2021). Based on this conceptualization, the United Nations recently launched the Decade of Healthy Ageing (2021–2030) with the goals of monitoring and optimizing older people’s functional ability for the benefit of both individuals and society (Michel et al. 2021). Meanwhile, other efforts have been made to construct common measures for healthy ageing to better compare results across cohorts and over time (Sanchez-Niubo et al. 2021). Although several scales measuring specific aspects of health and ageing have been developed, a comprehensive instrument for assessing IC and functional ability is still under debate (Bautmans et al. 2022). Nevertheless, Sanchez-Niubo et al. (2021) have used item-response theory to analyse relevant datasets from 16 international cohorts with the aim of developing a scale to assess healthy ageing that can be used globally, the Healthy Ageing Index (HAI) (Sanchez-Niubo et al. 2021). WHO has described six key domains of IC: vitality, visual capacity, hearing capacity, cognitive capacity, psychological capacity, and locomotor capacity (World Health Organization 2019). In this study, subjective age, objective and self-reported indicators covering health, functional ability and all six domains of IC suggested by WHO (World Health Organization 2019) are used to examine the relationship between subjective age and healthy ageing in a sample of individuals in Norway 60 years old or older.

## Materials and methods

### Participants

This research used data from the population-based Norwegian Survey of Health and Ageing (NORSE) (Strand et al. 2021), a study of health and living conditions conducted with a representative sample of the population 60 years old or above in the former Oppland County in Norway. The Norwegian Tax Administration gave permission to draw a random sample from the National Population Register. Three age strata were used: 60–69, 70–79 and 80+ years, with equal numbers drawn from each age group, achieving oversampling of the older age groups. Eligible participants were mailed by regular post a four-page leaflet and invitation letter with description of the study aims, testing procedures, and how data would be handled after the data collection. The leaflet contained ethical clearances and consent procedure, as well as how participants later could withdraw their consent at any time. Those willing to participate either sent a mobile text message or sign up using a pre-paid letter (Strand et al. 2021). Data were collected during 2017–2019. Out of 5981 invitations, a total of 957 participated. Descriptives of the sample were published in 2021 (Strand et al 2021). The 817 respondents with a valid response on the outcome variable assessing subjective age are included in the current analysis (14% response rate). Final-year nursing students at the Norwegian University of Science and Technology in Gjøvik, who were specially trained for the data collection, collected the data through standardized face to-face interviews, either at home or in local healthcare clinics or offices. Full population data from Oppland County for 2017 by age, sex, and level of education provided by Statistics Norway were used to create population weights to control for selection bias (Valliant and Dever 2018). This strategy provided us with information on the total population, including all nonrespondents, from administrative registries.

### Subjective age

Participants were asked if disregarding their actual age, how old did they feel. The absolute discrepancy, in years, between subjective and chronological age was calculated as subjective age minus chronological age. For example, a value of − 10 would indicate that the respondent was feeling 10 years younger than her chronological age.

### Indicators of IC

All six domains of IC were included in our study (1. Vitality, 2. Visual capacity, 3. Hearing capacity, 4. Cognitive capacity, 5. Psychological capacity, and 6. Locomotor capacity) (World Health Organization 2019). Because the aim was to investigate differences between groups, the study variables were dichotomized using established cut-off points.

#### Vitality

Vitality was assessed by hand grip strength (kg; Jamar hydraulic dynamometer; two attempts for each hand, including maximum score), self-reported chronic musculoskeletal pain (yes/no), degree of exhaustion (low/high), and incontinence (yes/no). Following the EWGSOP criteria (Cruz-Jentoft et al. 2010), grip strength scores were dichotomized as low (< 27 kg for men and < 16 kg for women) or high (≥ 27 kg for men and ≥ 16 kg for women).

#### Visual capacity

Visual capacity was based on self-reported vision assessed by the interview question “Is your eyesight [using glasses or contact lenses as usual]” 1. Excellent, 2. Good, 3. Fair, 4. Poor, or 5. I am blind (poor (3, 4 and 5/good (1 and 2)).

#### Hearing capacity

Hearing capacity was based on self-reported hearing assessed by the interview question “Is your hearing [using a hearing aid as usual]” 1. Excellent, 2. Very good, 3. Good, 4. Fair, 5. Poor (poor (4 and 5/good (1, 2 and 3)).

#### Cognitive capacity

Cognitive capacity was assessed with the Montreal Cognitive Assessment (MoCa) and grouped as normal (24–30), mild cognitive impairment (19–23), or dementia (0–18) (Carson, Leach, Murphy, 2018).

#### Psychological capacity

Psychological capacity was measured by assessments of depression, anxiety, and quality of sleep, using the EURO-D depression scale—no depressive symptoms (score 0–4), depressive symptoms (score 5–12) (Prince et al. 1999)—the generalized anxiety scale, GAD-7 (anxiety GAD ≥ 8, no anxiety GAD < 8) (Löwe, Decker et al. 2008), and self-reported sleep problems (yes/no), respectively.

#### Locomotor capacity

Locomotor capacity was assessed with the Short Physical Performance Battery (SPPB), in three groups rated as low performance (0–6), reduced performance (7–9), or normal performance (10–12) (Bergh, Lyshol et al. 2006).

#### Indicators of health

Health-related variables included self-reported general health (0 = very poor/poor/slightly poor; 1 = good/very good) and chronic disease (yes/no).

#### Indicators of functional ability, and frailty

Two variables on functional ability were included: frailty, using Fried’s criteria (Fried et al. 2001), and the widely used global activity limitation indicator (GALI) (Van Oyen, Van der Heyden et al. 2006, Berger, Van Oyen et al. 2015). The GALI was based on the question “For the past 6 months or more, have you been limited in activities people usually do because of a health problem?” and participants were grouped as 1 = Yes, strongly limited, 2 = Yes, limited, 3 = No, not limited. Fried's frailty criteria were based on all five original items: 1. Measured grip strength, 2. Measured gait speed (metre/second; based on the faster of two timed 4-m walks), 3. Self-reported weight loss, 4. Self-reported physical activity, and 5. Self-reported exhaustion.

### Statistical analysis

Mean and standard deviation of the outcome variable defined as the difference between subjective and chronological age was calculated for the total sample, and by categories such as sex, age groups, as well as for intrinsic capacity and health and function categories. We regressed the outcome variable against the intrinsic capacity, health and function variables, one by one, adjusted by sex and age. Furthermore, to account for nonresponses, the regression was weighted using inverse probability weighting and calibration. The inverse probability weights were constructed using Statistics Norway´s population for Oppland in 2017 by sex and age in five-year age groups (60–64, 65–69,70–74, 75–79, 80–84, 85+), and we assessed the size of each stratum in our study population. For example, in Oppland in 2017, there were 6032 women aged 60–64 years, while we had 100 of this group in our sample, which corresponds to 1.6%. Hence, each of these 100 women represented 6032/100 = 60.3 women. Thus, the weight 60.3 was assigned to all women aged 60–64 years. A similar procedure was applied for the other age and sex strata. In Stata, we used the *svyset* command, and inverse probability weighting (IPW) was used as the probability weight (pweight). Second, for calibration we used post-stratified weights to account for nonresponse bias due to education. We had access to the overall distribution of people in Oppland in 2017 by sex, in three age groups (60–69, 70–79, 80+) and at three educational levels (compulsory (< 10 years), secondary (10–12 years), and tertiary (13+ years)) from Statistics Norway. These data were merged with our data by matching strata and used as post-stratified weights. The reliability of the scales was investigated using Cronbach’s alpha.

## Results

**Fig. 1:**
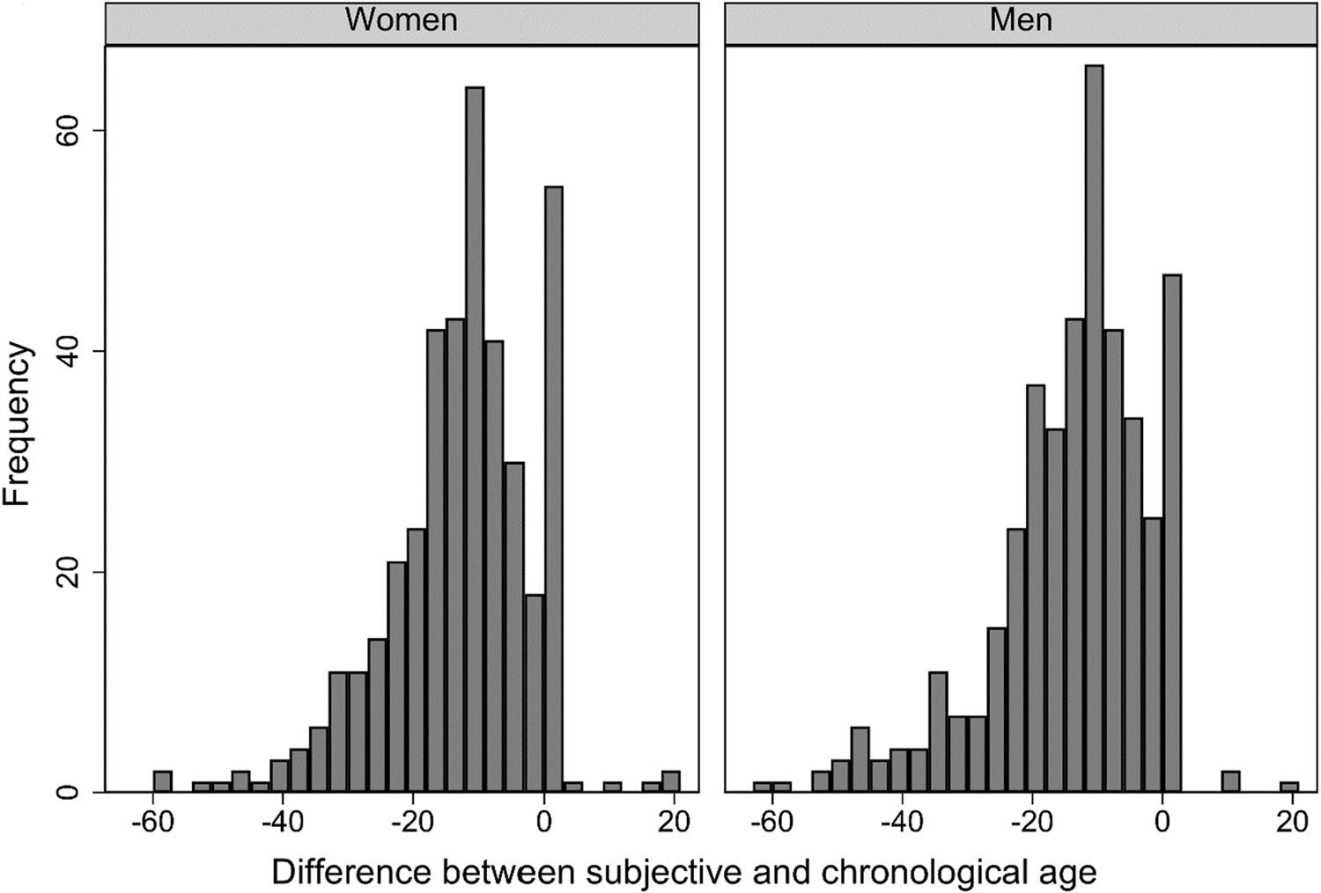
Frequency histogram, difference in years between subjective and chronological age, by sex. *N* = 817

In our sample, 86.5% felt younger than their chronological age, 5.2% felt older, and 8.3% felt exactly their chronological age (Fig. 1). These percentages were similar for men and women. Overall, the subjective age was found to be 13.8 years lower than chronological age (Table 1). This discrepancy between subjective and chronological age was slightly larger for men (14.3 years) than for women (13.2 years). It was also higher for the oldest chronological age group 80+ (15.2 years) compared to the youngest group aged 60–69 (13.5 years). However, the sex and age differences did not reach statistical significance in the weighted regression analyses and could have been due to chance (Table 1). Due to the similar discrepancy between subjective and chronological age between the sexes, men and women were collapsed in the analyses. With some exceptions, in age- and sex-adjusted analyses, there were few significant findings, but the tendency was in the expected direction, namely that poor health and poor function were associated with a smaller discrepancy between subjective and chronological age (Table 1). Among the IC indicators, those reporting having incontinence (vitality domain) the discrepancy between chronological and subjective age was 3.1 years narrower than those reporting no incontinence [95% confidence interval (CI) (0.6, 5.5)] adjusted by age and sex (Table 1). Correspondingly, for those with poor vision this difference was 2.9 years narrower compared to those not reporting poor vision [95% CI (0.2, 5.6)], and for those reporting poor hearing, this difference was 2.9 years narrower compared to those not reporting poor hearing [95% CI (0.3, 5.5)]. Some of the categories for vision, hearing and self-rated health had few responses. In addition to the analyses using dichotomized variables, we did finer analyses including the original scale, which also showed significant results and in the expected direction. There was no significant difference in the discrepancy between subjective and chronological age for cognitive capacity, psychological capacity, or locomotor capacity. For the health indicators, however, the discrepancy was 2.2 years narrower for those reporting poor health compared to those in good health (*p* = 0.076). No such discrepancies were found across the functional capacity or frailty indicators. Using metrical scales for grip strength, MoCa, EURO-D, GAD, SPPB, and frailty provided similar non-significant associations with the subjective age scores as in the analyses using dichotomized/categorical variables; in an age and sex adjusted, weighted analysis as in Table 1, using metrical scales the *p*-values were: grip strength 0.67, MoCa 0.56, EURO-D 0.66, GAD 0.43, SPPB 0.78, and frailty 0.22. Regarding the internal consistency, the Cronbach alpha were GAD = 0.78, EURO-D = 0.55, MoCa = 0.66, SPPB = 0.59, and frailty = 0.42. The overall internal consistency of the frailty scale was low. However, the frailty index is a multi-dimensional test including different components representing different constructs within two broader umbrella-constructs. The Cronbach’s alpha reliability coefficients of the frailty components ranged from 0.33 to 0.44, and average inter-item correlation ranged from 0.12 to 0.16. In an additional analysis, 26 subjective age scores were truncated ± 3 standard deviations apart from the mean. Results (not shown) were similar with and without this truncation and did not affect conclusions. Considering that we performed multiple testing (14 variables), the Bonferroni corrected *p*-value was 0.05/15 = 0.003. Using this conservative *p*-value as guidance for statistical significance, rather than the usual 0.05, none of the findings reached statistical significance.

**Table 1:** Descriptive statistics of the sample (*n* = 817) and results of the regression analysis investigating the association between the subjective and chronological age gap and intrinsic capacity, health and function

	*N*	Mean difference (in years) between subjective and chronological age	SD	Mean weighted* difference in years between subjective and chronological age, adjusted by age and sex, using one of the categories a reference	*p*-value	95% CI low	95% CI high
Total	817	− 13.77	11.64				
Men	418	− 14.29	11.96	− 0.8	0.363	− 2.6	1.0
Women	399	− 13.22	11.28	Ref	–	–	–
Age							
60–69	353	− 13.48	10.91	Ref	–	–	–
70–79	329	− 13.47	11.43	0.3	0.726	− 1.5	2.2
80+	135	− 15.24	13.78	− 2.3	0.128	− 5.2	0.7
*Intrinsic capacity*							
Vitality							
Grip strength							
High	716	− 13.81	11.15	Ref	–	–	–
Low	100	− 13.00	14.10	1.3	0.445	− 2.1	4.7
Musculoskeletal Pain							
No	286	− 13.33	10.80	Ref	–	–	–
Yes	529	− 14.02	12.10	− 1.0	0.299	− 2.9	0.9
Energetic							
No	557	− 13.83	10.99	Ref	–	–	–
Yes	255	− 13.70	13.01	0.8	0.449	− 1.3	2.8
Incontinent							
No	683	− 14.14	11.84	Ref	–	–	–
Yes	132	− 11.88	10.45	3.1**	0.015	0.6	5.5
Sensory function							
Vision							
Normal	711	− 14.07	11.49	Ref	–	–	–
Poor	103	− 11.56	12.53	2.9**	0.037	0.2	5.6
Hearing							
Normal	656	− 13.89	11.62	Ref	–	–	–
Poor	110	− 12.53	11.76	2.9**	0.030	0.3	5.5
Cognitive capacity							
(MoCa scores) Normal (24–30)	604	− 13.73	11.61	Ref	–	–	–
MCI (19–23)	171	− 13.35	11.43	1.8	0.100	− 0.3	3.9
Dementia (0–18)	37	− 17.41	13.10	− 1.8	0.445	− 6.5	2.9
Psychological capacity							
Depression (EURO-D)**							
No (0–4)	530	− 13.71	10.73	Ref	–	–	–
Yes (5–12)	287	− 13.87	13.18	0.7	0.510	− 1.3	2.6
Anxiety (GAD)**							
Normal (GAD < 8)	771	− 13.69	11.63	Ref	–	–	–
Anxiety (GAD ≥ 8)	46	− 14.98	11.89	0.1	0.956	− 4.0	4.3
Sleep problems							
No	563	− 13.69	11.44	Ref	–	–	–
Yes	253	− 13.94	12.12	− 0.4	0.720	− 2.5	1.7
Locomotor capacity							
SPPB score (0–12)							
Low (0–6)	53	− 15.17	16.77	Ref	–	–	–
Medium (7–9)	236	− 13.08	11.88	1.2	0.651	− 4.0	6.3
High (10–12)	528	− 13.93	10.90	0.3	0.906	− 4.8	5.5
*Health*							
Self-reported health							
Poor	181	− 12.40	13.95	Ref	–	–	–
Good	635	− 14.16	10.89	− 2.2	0.076	− 4.6	0.2
Chronic disease							
No	484	− 13.69	10.90	Ref	–	–	–
Yes	333	− 13.87	12.65	− 0.5	0.604	− 2.4	1.4
*Function*							
GALI							
No limitations	386	− 14.32	10.98	Ref	–	–	–
Some limitations	344	− 13.25	11.41	1.2	0.196	− 0.6	3.1
Substantial limitations	87	− 13.37	14.99	1.9	0.334	− 2.0	5.8
Frailty (Fried´s phenotype)							
Normal	400	− 13.78	10.71	Ref	–	–	–
Prefrail	260	− 14.00	11.42	− 0.4	0.698	− 2.1	1.4
Frail	106	− 14.10	13.89	3.6	0.121	− 0.9	8.1

Bonferroni *p*-value to account for multiple testing (15 tests): 0.003

*The regression was weighted to adjust for nonresponse due to age, sex and education; ** Significant results *p* < 0.05

## Discussion

Among home dwellers in Norway 60 years old or older in our sample, subjective age was consistently lower than chronological age, on average, by nearly 14 years. That finding aligns with past results (Stephan et al. 2013; Ye and Post 2020; Sayag and Kavé 2022; Veenstra et al. 2021). In prior research (Westerhof and Barrett 2005), those who felt younger than their chronological age were also found to generally have higher subjective well-being and positive emotions. The psychological pathways involved in subjective age can be complex and entail several reciprocal relationships, including with individual functional ability, health, and culture (Subramanian et al. 2009; Volz-Sidiropoulou and Gauggel 2012). Moreover, people may be able to sense changes in their physical health that have not yet been captured by objective health measures (Idler and Benyamini 1997) or they have been affected by negative views on ageing, which may potentially affect their subjective age and health behaviour (Wurm et al. 2017). This study found a substantially higher subjective age for participants who reported incontinence, poor vision, and poor hearing than for ones without those conditions, but other factors, including anxiety, depression, chronic disease, physical functional ability, cognition, and frailty, did not impact that discrepancy.

### Incontinence

To the best of our knowledge, this is the first study to investigate the association between incontinence and the discrepancy between subjective and chronological age, and it showed that participants with incontinence had a smaller discrepancy than those without the condition. Urinary continence is a common condition for both men and women (2004), its prevalence increases with age, and more than 40% of women 70 years old or older are affected (Milsom and Gyhagen 2019). Studies from the Swedish Twin Registry have presented evidence that genetic and nonshared environmental factors contribute equally to 40% of the variation in liability (Wennberg et al. 2011), and certain behaviours—childbirth, for example—can also affect the prevalence of incontinence (Waetjen et al. 2007). Incontinence is associated with loss of vitality (Sanchez-Niubo et al. 2021), embarrassment, and isolation (e.g. (Esparza et al. 2018)), as well as a lower quality of life (Pizzol et al. 2021). Beyond that, many men and women with the condition bear a significant mental health burden (Coyne et al. 2012). Considering past results, our novel finding suggests that incontinence needs to be addressed and acknowledged as a multifactorial public health concern.

### Vision loss, hearing loss and subjective age

Our study detected significantly smaller discrepancy between subjective and chronological age for those with poor vision and poor hearing than for the ones who did not report those sensory impairments. Although vision loss and hearing loss are common for older adults, few studies have investigated the association between the sensory functions of vision and hearing and subjective age. A recent study including 7085 individuals between 50 and 93 years of age from the Health and Retirement study found that subjective age was prospectively related to hearing function (Stephan, Sutin et al. 2022), but the studies that have been conducted have shown diverse findings. The German Ageing Survey, comprising 6378 individuals 40–89 years old observed over a nine-year period, showed that vision problems were associated with a higher subjective age (Wettstein et al. 2021a, b). However, no such result emerged in a relatively small study of 75 individuals, including a group of adults 93 years or older, but that study did find that hearing impairment was associated with higher subjective age (Schroyen et al. 2020).

### Grip strength, frailty, and depression

Grip strength, frailty, and depression were not significantly related with subjective age. This is in contrast to results from other studies (Debreczeni and Bailey 2021; Stephan et al. 2021). We can only speculate why the present results differ from those of these studies. It could also be that this setting is different, and that being depressed, less physically strong or frail does not relate to how old people see themselves, the present study population in the county of Oppland, Norway. It may be that many in this region are less likely to see physical or somatic change associated with age as relevant for how old they perceive themselves.

### Limitations

Among our study’s limitations, the sample came from only one region in Norway and may not be generalizable to other regions or countries. The study was also limited to an exclusive set of variables related to healthy ageing to investigate the association with subjective age, the evidence was cross-sectional, and the inclusion of confounding factors was not exhaustive. The sample was representative regarding sex and age but skewed towards a higher level of education, and likely prone to healthy selection bias. To account for this, our analyses were weighted by level of education to minimize selection biases. However, if the sample differed in factors other than those included, or the lower educated participants differ from the lower educated non-participants in functional ability and subjective age, the results may be biased, nonetheless. Limitations to this study includes the unknown causal directionality of the associations due to the cross-sectional study design, and that some of the measures only assess the presence of conditions and no other aspects such as the severity (for instance musculoskeletal pain). Our study’s overall response rate was low (14%), which also may have caused selection bias and a reduction in statistical power. Further, we used registry-based weights (for the whole population) to increase representativity. Last, our study is of limited size (*N* = 817), and for a range of sub-analyses and for properly addressing multiple testing, the groups were small (< 50). No effects remained significant after applying the conservative Bonferroni correction, so the analyses should be repeated in other, preferably larger, samples.

## Conclusion

In this study of adults aged 60+, feeling younger than ones’ chronological age is common. However, the discrepancy between subjective and chronological age was smaller for those who reported having incontinence, poor vision, or poor hearing than in participants without these conditions. Hence, higher subjective age may have undesirable relationships to vitality, autonomy, and perceived intrinsic capacity, which are considered imperative for healthy ageing.

## Data Availability

The NORSE project group can be contacted to get access to NORSE data. More information is found at the NORSE web site at the Norwegian Institute of Public Health: https://www.fhi.no/studier/norse-studien/norse/

## Appendix I: Correlation table of the included variables

**Table:**

	SEX	AGE	GRIP	PAIN	ENERGY	INCONT	VISION	HEARING	MOCA	EURO-D	GAD	SLEEP	SPPB	SRH	DIS	GALI	FRAIL
SEX	1																
AGE	− 0.0088	1															
GRIP	− 0.0489	0.2562*	1														
PAIN	− 0.1173*	− 0.0198	0.0852*	1													
ENERGY	− 0.1199*	0.1070*	0.1496*	0.1462*	1												
INCONT	− 0.1671*	0.1861*	0.0795*	0.1229*	0.1644*	1											
VISION	− 0.0922*	0.052	0.0916*	0.0742*	0.1051*	0.0875*	1										
HEARING	0.0397	0.1247*	0.0268	0.0581	0.1154*	0.0688	0.1149*	1									
MOCA	0.0707*	0.3181*	0.2088*	0.0697*	0.1632*	0.1230*	0.0579	0.1333*	1								
EURO-D	− 0.0957*	− 0.0111	0.0810*	0.0631	0.4141*	0.0387	0.1283*	0.0730*	0.0366	1							
GAD	− 0.1113*	0.0366	0.0219	0.1120*	0.2000*	0.1219*	− 0.0139	0.0699	0.1496*	0.1744*	1						
SLEEP	− 0.2413*	0.0692*	0.0471	0.1855*	0.2594*	0.2010*	0.0767*	0.0958*	0.0910*	0.063	0.1793*	1					
SPPB	0.0497	− 0.2926*	− 0.2174*	− 0.0838*	− 0.2284*	− 0.2409*	− 0.1115*	− 0.0814*	− 0.2944*	− 0.0940*	− 0.0663	− 0.1422*	1				
SRH	0.0146	− 0.0532	− 0.1755*	− 0.2091*	− 0.3646*	− 0.2072*	− 0.1381*	− 0.069	− 0.1558*	− 0.2039*	− 0.1117*	− 0.1832*	0.3298*	1			
DIS	− 0.0463	0.0395	0.1040*	0.2587*	0.2703*	0.1883*	0.0747*	0.0405	0.1111*	0.1452*	0.0990*	0.2109*	− 0.2355*	− 0.4463*	1		
GALI	− 0.0946*	0.1666*	0.2057*	0.2763*	0.3384*	0.2500*	0.1103*	0.1024*	0.2358*	0.1908*	0.1250*	0.1731*	− 0.3681*	− 0.5024*	0.5506*	1	
FRAIL	− 0.1230*	0.2760*	0.3811*	0.1788*	0.6583*	0.2469*	0.1698*	0.1093*	0.2744*	0.2886*	0.1689*	0.2160*	− 0.5116*	− 0.4041*	0.2988*	0.3908*	1

**p* < 0.05. Variables are identical to those presented in Table 1, in the same order
