# Correction: Subjective age and the association with intrinsic capacity, functional ability, and health among older adults in Norway

**DOI:** 10.1007/s10433-023-00757-y

**Published:** 2023-04-21

**Authors:** Ellen Melbye Langballe, Vegard Skirbekk, Bjørn Heine Strand

**Affiliations:** 1 Norwegian National Centre for Ageing and Health, Vestfold Hospital Trust, Tønsberg, Norway; 2 Department of Geriatric Medicine, Oslo University Hospital, Oslo, Norway; 3 Norwegian Institute of Public Health, Oslo, Norway

**Correction: European Journal of Ageing (2023) 20:4** 10.1007/s10433-023-00753-2

In the original publication of the article, the word “Urinary incontinence” should be replaced with “incontinence” throughout the article.

The original article has been corrected.

